# Shear Wave Elastography in Breast Cancer: Unveiling Correlations With Histopathological Grades and Subtypes

**DOI:** 10.7759/cureus.63759

**Published:** 2024-07-03

**Authors:** George Asafu Adjaye Frimpong, Evans Aboagye, Osei Owusu-Afriyie, Ernest O Bonsu, Fairuuj Mahama, Emmanuel Asante, Barima G Asafu Adjaye Frimpong

**Affiliations:** 1 Radiology, Kwame Nkrumah University of Science and Technology, Kumasi, GHA; 2 Radiology, Spectra Health Imaging and Interventional Radiology, Kumasi, GHA; 3 Research and Development, Spectra Health Imaging and Interventional Radiology, Kumasi, GHA; 4 Pathology, Kwame Nkrumah University of Science and Technology, Kumasi, GHA; 5 National Radiotherapy and Nuclear Medicine, Komfo Anokye Teaching Hospital, Kumasi, GHA; 6 Medicine, Kwame Nkrumah University of Science and Technology, Kumasi, GHA

**Keywords:** grade, b-mode ultrasound, histopathology, shear wave elastography, breast cancer

## Abstract

Objective

This study explores the correlation between shear wave elastography (SWE) features and histopathological grades and subtypes in breast cancer, aiming to enhance diagnostic accuracy and personalized treatment strategies.

Methods

The study retrospectively analyzed 59 consecutive women with breast cancer who underwent breast ultrasound with SWE. SWE parameters and histopathologic information, including histological type and grade, were recorded. Qualitative and quantitative SWE findings were analyzed, and B-mode findings were evaluated. Sociodemographic and clinical factors and B-mode findings were assessed as predictors of elastography stiffness using logistic regression analysis.

Results

Of the 59 participants diagnosed with breast cancer, invasive ductal carcinoma of no special type (IDC-NST) was predominantly found in 50 (84.7%) cases, followed by invasive medullary carcinoma in 5 (8.5%) cases. The majority of participants belonged to the 50-59 age group, comprising 19 (32.2%) patients. Histopathological grading revealed grade II tumors in 27 (45.8%) cases and grade III tumors in 24 (40.7%) cases. Notably, grade III tumors exhibited higher tissue stiffness compared to grade II tumors. Out of 36 stiff lesions, 30 (83.3%%) were IDC-NST while 3 (8.3%) were invasive medullary carcinoma. A significant association was observed between higher histopathological grade (grade III) and increased tissue stiffness (p < 0.05). Furthermore, among participants with stiff lesions, 21 (58.3%) exhibited color defects while 4 (23.5%) cases with soft lesions also displayed color defects

Conclusion

The correlation between SWE findings and histopathological grades and subtypes underscores the potential of SWE as a valuable tool for predicting tumor aggressiveness and characterizing specific subtypes. SWE enhances diagnostic accuracy and complements traditional imaging modalities, holding promise for personalized treatment strategies.

## Introduction

Breast cancer remains a significant global health challenge, with substantial morbidity and mortality rates [1]. Despite advances in imaging technologies, the early and accurate characterization of breast tumors remains crucial for optimal patient management and treatment outcomes. Traditional imaging methods like ultrasound and mammography play a pivotal role in the initial detection and assessment of breast lesions; however, they often fall short of precisely characterizing the biological behavior of tumors [2]. As a result, there is a need for additional tools that can enhance diagnostic accuracy and aid in personalized treatment strategies.

Shear wave elastography (SWE) has emerged as a promising adjunctive tool in the field of breast cancer evaluation. Unlike traditional imaging modalities that rely on anatomical features, SWE provides quantitative and qualitative information about tissue stiffness. It utilizes shear waves generated by an acoustic radiation force to assess the mechanical properties of tissues [3]. Tumors exhibit altered biomechanical properties compared to normal breast tissue due to changes in cellularity, extracellular matrix composition, and tissue architecture [4]. Consequently, SWE can provide valuable insights into tumor aggressiveness and aid in characterizing specific subtypes [5].

Histopathological grading and subtyping are crucial aspects of breast cancer diagnosis and prognosis. They provide valuable information about tumor behavior, guide treatment decisions, and influence patient outcomes [6]. However, obtaining histopathological information typically requires invasive procedures such as biopsies or surgical excisions. SWE, as a non-invasive imaging modality, has the potential to complement histopathological assessments by providing additional information about tumor stiffness and heterogeneity [7].

Despite its potential, the application of SWE in clinical practice varies widely, with inconsistent integration into diagnostic protocols. This variability stems partly from a lack of comprehensive understanding of how SWE findings correlate with established histopathological grades and subtypes of breast cancer, which are crucial for determining prognosis and tailoring treatment plans [8,9]. Moreover, most existing studies have focused on the diagnostic accuracy of SWE rather than its predictive capacity regarding tumor behavior and response to treatment.

This study aims to fill these gaps by analyzing the correlations between SWE features and histopathological grades and the subtypes of breast cancer. We sought to elucidate the potential of SWE to enhance diagnostic precision and contribute to more personalized treatment strategies, thereby improving patient outcomes. Our findings aim to provide a foundation for integrating SWE into routine diagnostic workups, ensuring that patients benefit from more informed treatment choices based on a non-invasive assessment of tumor stiffness.

## Materials and methods

Study design and participants

This retrospective study aimed to uncover the correlations between shear wave elastography (SWE) features and histopathological grades and subtypes in breast cancer patients. We reviewed 59 medical records and imaging data of consecutive women diagnosed with breast cancer who underwent breast imaging as part of their diagnostic evaluation at Spectra Health and Imaging and Interventional Radiology between June 2020 and December 2023. The inclusion criteria were adult women (age ≥ 18 years) who had received both conventional breast imaging (mammography and/or ultrasound) and SWE prior to any therapeutic intervention. Patients with breast implants, prior breast surgery, or those lacking complete imaging or histopathological data were excluded from the study.

Shear wave elastography technique

**Figure 1:**
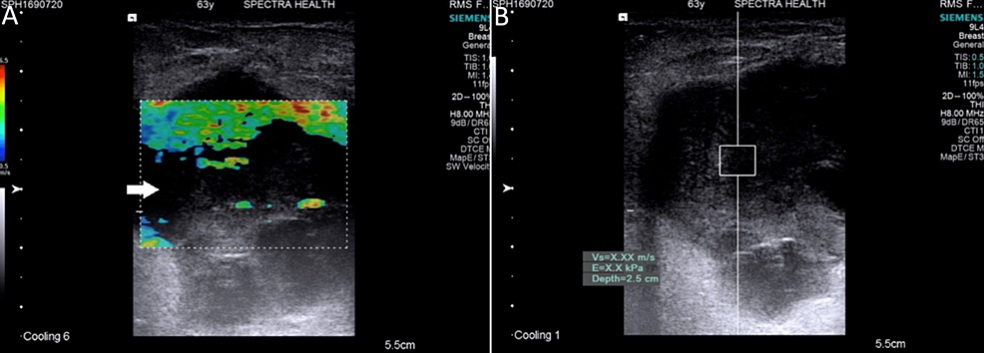
Malignant elastography images with color-filling defect (invasive carcinoma, nonspecific type (IC, NST) Grade 2) (A) Qualitative elastography shows low stiffness with a predominantly green color map and a large area of color-filling defects (arrow); (B) Quantitative elastography shows very high stiffness with ARFI velocity beyond the measurable range recorded as X.XX m/s (≥6.0 m/s) ARFI: acoustic radiation force impulse

**Figure 2:**
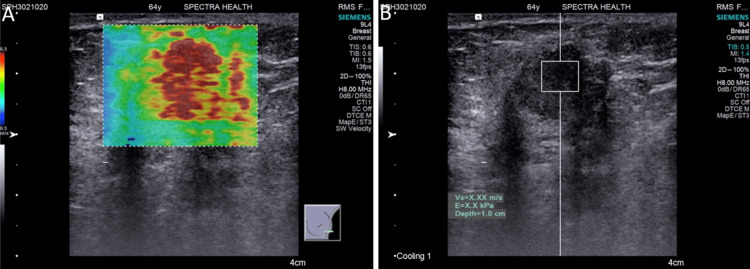
Malignant elastography images without color filling defect (Invasive micropapillary carcinoma (IMC), Grade 3): (A) Qualitative elastography shows very high stiffness with a predominantly red color map; (B) Quantitative elastography shows very high stiffness with ARFI velocity beyond the measurable range recorded as X.XX m/s (≥6.0 m/s).

All SWE examinations were performed using the Siemens Acuson S3000 Ultrasound System (Siemens Medical Solutions USA, Inc., PA, USA), equipped with a high-frequency linear transducer (7-12 MHz). Patients were examined in the supine position with their arms elevated. SWE was conducted alongside traditional B-mode ultrasound to provide a comprehensive lesion assessment. The SWE parameters recorded included Virtual Touch Imaging (VTI™️) and Virtual Touch Quantification (VTQ™️) values (Figures 1, 2). VTQ was obtained as shear wave velocity (SWV) by placing a region of interest (ROI) entirely in the breast lesion on the US display without including the adjacent parenchyma. The upper limit of SWV displayed on the ultrasound scanner utilized in our study was 6.0 m/s. When SWV values exceeded this limit, they were represented as X.XX, denoting SWV ≥6.0 m/s after mitigating factors such as the patient’s respiration, movement, and inappropriate placement of the ROI. Two experienced radiologists, each with over 15 years of sonography experience and 5 years specifically with SWE, conducted and independently reviewed all examinations.

Data collection

Data extracted from medical records included patient demographics, clinical history, histological type of cancer, and histological grade. The primary variables of interest from the SWE included quantitative shear wave velocities (measured in meters per second, m/s) and qualitative assessments of tissue stiffness.

Data analysis

Data were summarized using descriptive statistics, with means and standard deviations calculated for continuous variables and frequencies and percentages for categorical variables. The association between SWE parameters and histopathological features was analyzed using chi-square tests for categorical variables and t-tests or analysis of variance (ANOVA) for continuous variables, as appropriate. Multivariable logistic regression analyses were conducted to adjust for potential confounders, such as age and hormone receptor status, with stiffness as the dependent variable and histopathological grade and subtype as independent variables. Statistical significance was set at a p-value of less than 0.05. All statistical analyses were performed using SPSS version 25 (IBM Corp., Armonk, NY, USA).

Ethical considerations

The study protocol was reviewed and approved by the Committee on Human Research, Publication, and Ethics (CHRPE) at the Kwame Nkrumah University of Science and Technology (CHRPE/RC/028/20). All patients waived informed consent for their data to be used for research purposes, and all patient information was anonymized and de-identified prior to analysis to ensure confidentiality.

## Results

Baseline characteristics of study participants

This study included 59 participants, with a mean age of 54.58±11.96 years. The majority of participants (19; 32.2%), were in the 50-59 age range, followed by the 60-69 and 40-49 age ranges, each comprising 14 (23.7%) participants. The smallest group, consisting of 5 (8.5%) participants, fell within the 20-39 age range. Histological analysis revealed that invasive ductal carcinoma of no special type (IDC-NST) was the most prevalent cancer type, observed in 50 (84.7%) cases. This was followed by invasive medullary carcinoma, which was present in 5 (8.5%) participants. In terms of histologic grade, 27 (45.8%) participants had grade II lesions, followed by grade III, 24 (40.7%). The detailed distribution is shown in Table 1.

**Table 1:** Baseline characteristics of study participants

Variable	Frequency (n=59)
Age (Years) (π ±SD)	54.58 ±11.96
Age Group (Years)	
20-39	5 (8.5%)
40-49	14 (23.7%)
50-59	19 (32.2%)
60-69	14 (23.7%)
70-76	7 (11.9%)
Histology	
Low-grade sarcoma	1 (1.7%)
Metastasis from granulosa cell tumor	1 (1.7%)
Ductal carcinoma in situ (DCIS)	1 (1.7%)
Invasive lobular carcinoma (ILC)	1 (1.7%)
Invasive medullary carcinoma (IMC)	5 (8.5%)
Invasive ductal carcinoma, no special type (IDC-NST)	50 (84.7%)
Histology Grade	
Grade 1	8 (13.6%)
Grade II	27 (45.8%)
Grade III	24 (40.7%)

Qualitative and quantitative findings of shear wave elastography

**Figure 3:**
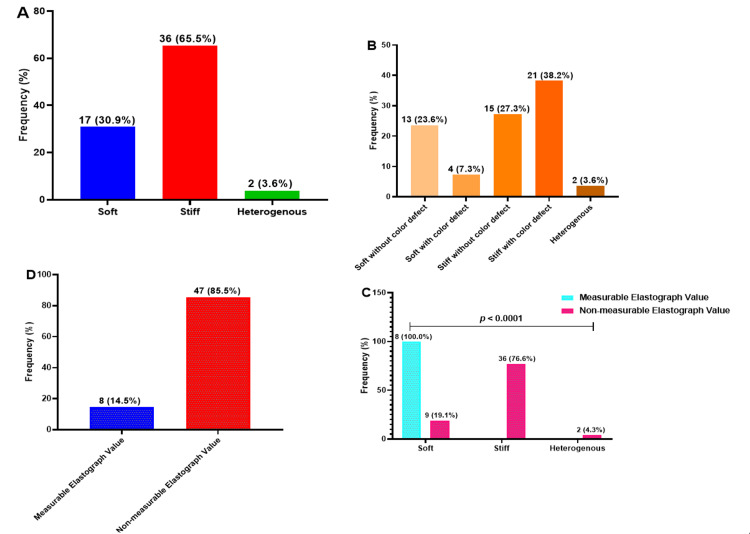
Qualitative (A-B) and quantitative (D-C) findings of elastography

The qualitative and quantitative findings of SWE revealed that 36 (65.5%) participants had stiff lesions while 17 (30.9%) had soft lesions. Among those with stiff lesions, 21 (58.3%) had color defects, whereas 15 (41.7%) did not. In contrast, among those with soft lesions, 4 (23.5%) had color defects while 13 (76.5%) did not (Figures 1, 2). Most participants (47; 85.5%), had non-measurable elastography values, indicating shear wave velocities ≥6 m/s while 8 (14.5%) participants had measurable elastography values, all found exclusively in participants with soft lesions. The comparison between qualitative and quantitative measurements demonstrated a significant difference, p <0.05 (Figure 3).

B-mode findings pattern of breast cancer patients

The study observed that among the participants, 36 (61%) had well-defined lesions while 19 (32.2%) had ill-defined lesions. The majority of the lesions were lobulated, accounting for 19 (32.2%) cases, while 10 (16.9%) exhibited architectural distortion. Also, in 10 (16.9%) cases, lesions were taller than wide, in 8 (13.6%) cases, lesions were round, while in 7 (11.9%), lesions were oval. Regarding the internal characteristics, 49 (83.1%) lesions were heterogeneous. Vascularity assessment showed that 29 (49.2%) lesions had mild vascularity and 19 (32.2%) lesions displayed moderate vascularity. Approximately one-third of the lesions, specifically 18 (30.5%), were calcified (Table 2).

**Table 2:** B-mode findings pattern of breast cancer patients

Variable	Frequency (n=59)
Lesion Boundary	
Well-defined	36 (61.0%)
Speculated	4 (6.8%)
Ill-defined	19 (32.2%)
Lesion Shape	
Oval	7 (11.9%)
Round	8 (13.6%)
Taller than wide	10 (16.9%)
Wider than tall	5 (8.5%)
Architectural distortion	10 (16.9%)
Lobulated	19 (32.2%)
Lesion Homogeneity	
Homogenous	10 (16.9%)
Heterogenous	49 (83.1%)
Vascularity	
Negative	8 (13.6%)
Mild vascularity	29 (49.2%)
Moderate vascularity	19 (32.2%)
Marked vascularity	3 (5.1%)
Calcifications	
Negative	41 (69.5%)
Positive	18 (30.5%)

Sociodemographic, clinical factors, and B-mode findings patterns as predictors of elastography stiffness

In a logistic regression prediction model, the study found that ill-defined lesions and heterogeneous lesions were significantly associated with a six-fold increase in stiffness among the study participants. Specifically, ill-defined lesions had an odds ratio (cOR) of 6.18, with a 95% confidence interval (CI) of 1.20-31.72 and a p-value of 0.029. Heterogeneous lesions had a cOR of 5.82, with a 95% CI of 1.24-27.30 and a p-value of 0.026. Although not statistically significant, calcifications and lobulated lesions showed an increased likelihood of stiffness among the study participants. Calcifications had a cOR of 4.77, with a 95% CI of 0.94-24.13 and a p-value of 0.059, while lobulated lesions had a cOR of 4.00, with a 95% CI of 0.39-41.23 and a p-value of 0.244.

Similarly, grade II and grade III lesions were associated with increased chances of stiffness among the study participants. However, these associations were not statistically significant. Grade II lesions had a cOR of 1.37, with a 95% CI of 0.25-7.39 and a p-value of 0.713, while grade III lesions had a cOR of 1.56, with a 95% CI of 0.27-9.11 and a p-value of 0.621. Mild lesions had a cOR of 3.78, with a 95% CI of 0.65-22.02 and a p-value of 0.139, while moderate lesions had a cOR of 2.22, with a 95% CI of 0.37-13.54 and a p-value of 0.386. The findings are presented in Table 3.

**Table 3:** Sociodemographic, clinical factors and B-mode findings patterns as predictors of elastography stiffness p-values computed by the logistics regression prediction model, p-values < 0.05 and bolded means statistically significant

Variable	cOR (95% CI)	p-value
Age (Years) (π ±SD)	0.98 (0.93-1.03)	0.448
Age Group (Years)		
20-39	1.00	-
40-49	1.50 (0.18-12.78)	0.711
50-59	4.67 (0.46-47.63)	0.194
60-69	0.78 (0.10-6.32)	0.814
70-76	0.67 (0.06-7.35)	0.741
Histology		
Low-grade sarcoma	1.00	-
Metastasis from granulosa cell tumor	1.00 (0.00-inf)	> 0.999
Ductal carcinoma in situ (DCIS)	0.00 (0.00-inf)	> 0.999
Invasive lobular carcinoma (ILC)	1.00 (0.00-inf)	> 0.999
Invasive medullary carcinoma (IMC)	0.00 (0.00-inf)	> 0.999
Invasive ductal carcinoma, no special type (IDC NST)	0.00 (0.00-inf)	> 0.999
Histology Grade		
Grade 1	1.00	-
Grade II	1.37 (0.25-7.39)	0.713
Grade III	1.56 (0.27-9.11)	0.621
Lesion Boundary		
Well-defined	1.00	-
Speculated	2.47 (0.23-26.46)	0.455
Ill-defined	6.18 (1.20-31.72)	0.029
Lesion Shape		
Oval	1.00	-
Round	0.00 (0.00-inf)	0.999
Taller than wide	1076983229.00 (0.00-inf)	0.999
Wider than tall	0.67 (0.03-18.06)	0.810
Architectural distortion	1076983229.00 (0.00-inf)	0.999
Lobulated	4.00 (0.39-41.23)	0.244
Lesion Homogeneity		
Homogenous	1.00	-
Heterogenous	5.82 (1.24-27.30)	0.026
Vascularity		
Negative	1.00	-
Mild vascularity	3.78 (0.65-22.02)	0.139
Moderate vascularity	2.22 (0.37-13.54)	0.386
Marked vascularity	2153966457.00 (0.00-inf)	0.999
Calcifications		
Negative	1.00	-
Positive	4.77 (0.94-24.13)	0.059

Association between sociodemographic, clinical factors, histologic findings, and elastography findings

The study found that participants with invasive ductal carcinoma of no special type (IDC-NST) mainly exhibited stiff lesions, with color defects in 20 (95.2%) cases and without color defects in 10 (66.7%) cases. On the other hand, participants with invasive medullary carcinoma consistently displayed elastography findings without color defects; 3 (20.0%) of stiff lesions and 2 (15.4%) of soft lesions. However, the study did not establish a significant difference between the histological presentations and elastography findings among the participants (p = 0.427). The findings are presented in Table 4.

**Table 4:** Association between sociodemographic, clinical factors, histologic findings, and elastography findings ^a^p-values computed by one-way ANOVA, ^b^p-values computed by the chi-square test, n/c: p-values not computed due to constant, p-values < 0.05, and bolded means statistically significant

Variable	Total (n=53)	Soft without Color Defect (n=13)	Soft with Color Defect (n=4)	Stiff without Color Defect (n=15)	Stiff with Color Defect (n=21)	p-value
Age (Years) (π ±SD)	54.58 ±11.96	54.54 ±13.44	60.25 ±18.75	54.27 ±12.45	52.43 ±10.05	0.705^a^
Age Group (Years)	-	-	-	-	-	0.339^b^
20-39	5 (9.4)	1 (7.7)	1 (25.0)	1 (6.7)	2 (9.5)	-
40-49	13 (24.5)	4 (30.8)	0 (0.0)	4 (26.7)	5 (23.8)	-
50-59	16 (30.2)	2 (15.4)	0 (0.0)	5 (33.3)	9 (42.9)	-
60-69	13 (24.5)	5 (38.5)	1 (25.0)	3 (20.0)	4 (19.0)	-
70-76	6 (11.3)	1 (7.7)	2 (50.0)	2 (13.3)	1 (4.8)	-
Histology	-	-	-	-	-	0.427^b^
Low-grade sarcoma	1 (1.9)	0 (0.0)	0 (0.0)	1 (6.7)	0 (0.0)	-
Metastasis from granulosa cell tumor	1 (1.9)	0 (0.0)	0 (0.0)	1 (6.7)	0 (0.0)	-
Ductal carcinoma in situ (DCIS)	1 (1.9)	1 (7.7)	0 (0.0)	0 (0.0)	0 (0.0)	-
Invasive lobular carcinoma (ILC)	1 (1.9)	0 (0.0)	0 (0.0)	0 (0.0)	1 (4.8)	-
Invasive medullary carcinoma (IMC)	5 (9.4)	2 (15.4)	0 (0.0)	3 (20.0)	0 (0.0)	-
Invasive ductal carcinoma, no special type (IDC-NST)	44 (83.0)	10 (76.9)	4 (100.0)	10 (66.7)	20 (95.2)	-
Histology Grade	-	-	-	-	-	0.960^b^
Grade 1	8 (16.3)	2 (16.7)	1 (33.3)	2 (15.4)	3 (14.3)	-
Grade II	23 (46.9)	6 (50.0)	1 (33.3)	5 (38.5)	11 (52.4)	-
Grade III	18 (36.7)	4 (33.3)	1 (33.3)	6 (46.2)	7 (33.3)	-

## Discussion

The present study aimed to investigate the correlations between SWE features and histopathological grades and subtypes in breast cancer. The findings of this study contribute to the growing body of evidence supporting the diagnostic utility of SWE in breast cancer evaluation and provide valuable insights into tumor aggressiveness and the characterization of specific subtypes.

Consistent with previous studies, our results demonstrated a significant association between higher histopathological grade (grade III) and increased tissue stiffness as measured by SWE. This finding is in line with the findings of Chang et al. [8] and Gemici et al. [9], who also reported a positive correlation between tumor grade and SWE stiffness values. The observed association between a higher histopathological grade and increased tissue stiffness can be explained by the underlying biological changes in breast tumors. High-grade tumors often exhibit more aggressive characteristics, including increased cellularity, higher mitotic activity, and a higher proportion of stromal components. These factors contribute to increased tissue stiffness, as reflected by SWE measurements [10]. The non-invasive nature of SWE makes it a valuable tool for assessing tumor aggressiveness without the need for invasive procedures such as biopsies or surgical interventions.

Our study revealed that invasive ductal carcinoma of no special type had a higher proportion of stiff elastography values compared to invasive medullary carcinoma. This observation is consistent with the findings of Kang et al. [11], who reported that invasive ductal carcinoma subtypes exhibited higher SWE stiffness values compared to other subtypes. These consistent findings across studies provide robust evidence of the potential of SWE to distinguish between different subtypes of breast cancer based on their biomechanical properties. The observed association between higher SWE stiffness values in invasive ductal carcinoma of no special type (IDC-NST) can be attributed to the underlying differences in the tumor biology and microenvironment. IDC-NST is known to be associated with higher cellularity, increased stromal components, and more aggressive tumor behavior [12]. These factors contribute to increased tissue stiffness, which is reflected in higher SWE measurements. The ability of SWE to differentiate between different histological subtypes based on their biomechanical properties is of significant clinical relevance. Histological subtyping is an important aspect of breast cancer diagnosis and treatment planning, as different subtypes may have distinct clinical behaviors and treatment responses [13]. By providing additional information on the biomechanical properties of tumors, SWE can potentially enhance the accuracy of subtype classification, leading to more personalized treatment strategies.

Also, qualitative elastography analysis revealed that a significant proportion of participants with stiff lesions exhibited color defects. This finding suggests that color defects in elastography images may serve as a qualitative marker, potentially indicative of areas of increased tissue stiffness. The presence of color defects could be attributed to alterations in tissue composition or vascularity within the lesions. The identification of such defects could be crucial for recognizing variations within the subtypes of breast cancers, providing valuable insights for further diagnostic and therapeutic considerations [14]. This information highlights the potential of qualitative elastography analysis as an adjunctive tool for identifying potentially malignant or abnormal tissue areas [15].

In addition, our findings revealed significant associations between ill-defined and heterogeneous lesions with increased stiffness in breast lesions. The presence of calcifications and lobulated lesions also showed a trend toward higher likelihoods of stiffness. Histopathological factors, such as higher grades and moderate/mild lesions, exhibited increased chances of stiffness, although not statistically significant. These findings emphasize the potential of incorporating these factors in predicting elastography stiffness and provide valuable insights for improving the interpretation and clinical utility of elastography in breast lesion evaluation. The trend observed in this study aligns with the existing knowledge on the relationship between histopathological features and tissue stiffness [16,17].

The integration of SWE into clinical practice has the potential to enhance diagnostic accuracy in breast cancer evaluation [8]. Traditional imaging modalities, such as ultrasound and mammography, have limitations in providing detailed information about tumor characteristics and aggressiveness [2]. In contrast, SWE provides quantitative information about tissue stiffness, which is directly related to the biomechanical properties of tumors. The combination of SWE with traditional imaging modalities offers a comprehensive evaluation of breast lesions. While ultrasound and mammography provide information about the anatomical features of the lesion, SWE adds a functional dimension by assessing tissue stiffness [18]. This integration allows for a more accurate characterization of lesions and can aid in distinguishing between benign and malignant tumors [19].

Despite the promising results, it is important to acknowledge the limitations of our study. The sample size was relatively small, which may limit the generalizability of the findings. Future studies with larger sample sizes are needed to validate the results and further explore the potential of SWE in breast cancer evaluation. Additionally, our study was retrospective in nature, introducing the possibility of selection bias. Prospective studies are warranted to confirm the correlations observed in this study and to evaluate the predictive value of SWE in larger patient cohorts.

## Conclusions

This study substantiates the potential of shear wave elastography (SWE) as a pivotal diagnostic tool in breast cancer assessment. By correlating SWE findings with histopathological grades and subtypes of malignant tumors, we demonstrated that SWE not only enhances diagnostic accuracy but also provides valuable insights into tumor aggressiveness and heterogeneity. The significant associations between increased tissue stiffness and higher histopathological grades underscore the utility of SWE in non-invasively predicting tumor characteristics, which can be instrumental in tailoring personalized treatment strategies. Integrating SWE into clinical protocols used in African populations could substantially improve patient management by facilitating more precise and early interventions based on detailed, real-time tissue characterization. Further research will be crucial to explore the full clinical implications and refine the application of SWE in diverse patient populations.

## References

[1] Barrios CH (2022). Global challenges in breast cancer detection and treatment. Breast.

[2] Lima ZS, Ebadi MR, Amjad G, Younesi L (2019). Application of imaging technologies in breast cancer detection: a review article. Open Access Maced J Med Sci.

[3] Papageorgiou I, Valous NA, Hadjidemetriou S, Teichgräber U, Malich A (2022). Quantitative assessment of breast-tumor stiffness using shear-wave elastography histograms. Diagnostics (Basel).

[4] Fenner J, Stacer AC, Winterroth F, Johnson TD, Luker KE, Luker GD (2014). Macroscopic stiffness of breast tumors predicts metastasis. Sci Rep.

[5] Buonomo OC, Caredda E, Portarena I (2017). New insights into the metastatic behavior after breast cancer surgery, according to well-established clinicopathological variables and molecular subtypes. PLoS One.

[6] Malhotra GK, Zhao X, Band H, Band V (2010). Histological, molecular and functional subtypes of breast cancers. Cancer Biol Ther.

[7] Elyas E, Papaevangelou E, Alles EJ, Erler JT, Cox TR, Robinson SP, Bamber JC (2017). Correlation of ultrasound shear wave elastography with pathological analysis in a xenografic tumor model. Sci Rep.

[8] Chang JM, Park IA, Lee SH (2013). Stiffness of tumours measured by shear-wave elastography correlated with subtypes of breast cancer. Eur Radiol.

[9] Gemici AA, Ozal ST, Hocaoglu E, Inci E (2020). Relationship between shear wave elastography findings and histologic prognostic factors of invasive breast cancer. Ultrasound Q.

[10] Yoo J, Seo BK, Park EK (2020). Tumor stiffness measured by shear wave elastography correlates with tumor hypoxia as well as histologic biomarkers in breast cancer. Cancer Imaging.

[11] Kang HJ, Kim JY, Lee NK, Lee JW, Song YS, Park SY, Shin JK (2018). Three-dimensional versus two-dimensional shear-wave elastography: associations of mean elasticity values with prognostic factors and tumor subtypes of breast cancer. Clin Imaging.

[12] Makki J (2015). Diversity of breast carcinoma: histological subtypes and clinical relevance. Clin Med Insights Pathol.

[13] Masood S (2016). Breast cancer subtypes: morphologic and biologic characterization. Womens Health (Lond).

[14] Balleyguier C, Ciolovan L, Ammari S (2013). Breast elastography: the technical process and its applications. Diagn Interv Imaging.

[15] Kim HJ, Kim SM, Kim B (2018). Comparison of strain and shear wave elastography for qualitative and quantitative assessment of breast masses in the same population. Sci Rep.

[16] Zhou J, Zhan W, Chang C (2014). Breast lesions: evaluation with shear wave elastography, with special emphasis on the "stiff rim" sign. Radiology.

[17] Durhan G, Öztekin PS, Ünverdi H (2017). Do histopathological features and microcalcification affect the elasticity of breast cancer?. J Ultrasound Med.

[18] Sigrist RM, Liau J, Kaffas AE, Chammas MC, Willmann JK (2017). Ultrasound elastography: review of techniques and clinical applications. Theranostics.

[19] Han Z, Li J, Singh M (2015). Quantitative methods for reconstructing tissue biomechanical properties in optical coherence elastography: a comparison study. Phys Med Biol.

